# Protective Effect of *Lycium ruthenicum* Murr. Against Radiation Injury in Mice

**DOI:** 10.3390/ijerph120708332

**Published:** 2015-07-17

**Authors:** Yabin Duan, Fan Chen, Xingchen Yao, Junbo Zhu, Cai Wang, Juanling Zhang, Xiangyang Li

**Affiliations:** 1 Department of Pharmacy, Qinghai University Medical College, Xining 810001, China; E-Mails: ddpatentdd@163.com (Y.D.); yaoxingcheng0625@163.com (X.Y.); qhmczjb@163.com (J.Z.); qhdxzjl@sina.com (J.Z.); 2 Department of Radiotherapy Oncology, Qinghai University Affiliated Hospital, Xining 810001, China; E-Mails: chfa1964@126.com (F.C.); jiayun_660@163.com (C.W.)

**Keywords:** *Lycium ruthenicum* Murr., radioprotection, apoptosis, hematopoietic

## Abstract

The protective effect of *Lycium ruthenicum* Murr. against radiation injury was examined in mice. Kunming mice were randomly divided into a control group, model group, positive drug group and *L. ruthenicum* high dose (8 g/kg), *L. ruthenicum* middle dose (4 g/kg), *L. ruthenicum* low dose (2 g/kg) treatment groups, for which doses were administered the third day, seventh day and 14th day after irradiation. *L. ruthenicum* extract was administered orally to the mice in the three treatment groups and normal saline was administered orally to the mice in the control group and model group for 14 days. The positive group was treated with amifostine (WR-2721) at 30 min before irradiation. Except for the control group, the groups of mice received a 5 Gy quantity of X-radiation evenly over their whole body at one time. Body weight, hemogram, thymus and spleen index, DNA, caspase-3, caspase-6, and P53 contents were observed at the third day, seventh day, and 14th day after irradiation. *L. ruthenicum* could significantly increase the total red blood cell count, hemoglobin count and DNA contents (*p <* 0.05). The spleen index recovered significantly by the third day and 14th day after irradiation (*p <* 0.05)*. L. ruthenicum* low dose group showed a significant reduction in caspase-3 and caspase-6 of serum in mice at the third day, seventh day, and 14th day after irradiation and *L. ruthenicum* middle dose group experienced a reduction in caspase-6 of serum in mice by the seventh day after irradiation. *L. ruthenicum* could decrease the expression of P53. The results showed that *L. ruthenicum* had protective effects against radiation injury in mice.

## 1. Introduction

Ionizing radiation has become one of the most significant threats to human health. Ionizing radiation as well as water, air, and noise pollution are regarded as four major kinds of pollution in the world. All tissues and organs can be damaged while the blood system, reproductive system, and digestive system are the more radiation-sensitive target organs. At present, the main drug therapies are toxic to the healthy cells, which causes other negative effects. Amifostine (WR-2721), developed the by U.S. Army Research Institute, can significantly protect normal cells and reduce apoptosis after radiation therapy, and it is the only cytoprotective agent approved by the Food and Drug Administration (FDA) specifically for use as a radioprophylactic [1,2]. However, low blood pressure, nausea, vomiting and other adverse reactions have still restricted its broad use [3,4,5]. Therefore, the search for anti-radiation drugs is one of the most important tasks, which could have a positive effect on patients after radiation therapy. Natural chemicals have the advantages of low toxicity, wide effects and so on. They can be applied to many target organs and can reduce radiation damage Therefore, it is important to search for high efficiency, low toxicity, radioprotective agents from natural products. Now, research on radiation protectants has gradually expanded from the previous synthetic-compound chemicals to natural anti-radiation drugs and functional foods. Scholars have already achieved some results [6,7,8].

*Lycium ruthenicum* Murr. belongs to Solanaceae Lycium, and is widely distributed in the salinized desert of the Qinghai-Tibet Plateau. It has been recorded in Tibetan medical classic “Jing Zhu Ben Cao” [9]. *L. ruthenicum* is not only a kind of Chinese herb, but also a unique nutritional food with plenty of proanthocyanidins, anthocyanins and polysaccharides [10,11,12]. A toxicological assessment of pigments of *L. ruthenicum* has been undertaken, which found *L. ruthenicum* has good edible-safety properties which means it can be widely used as a natural food plant-pigment [13]. Previous reports indicated that *L. ruthenicum* can effectively eliminate free radicals, with anti-oxidation and anti-aging efficiency [14,15,16]. However, up to now, no comprehensive study has been conducted to explore the anti-radiation effects of *L. ruthenicum.*

The morbidity of cancer has increased in recent years and many patients need to receive chemotherapy and radiation therapy. Radiation therapy is still the major treatment modality, however, it results in suppression of hematopoiesis accompanied by radiation-induced apoptosis of bone marrow cells. Patients find it hard to bear the side-effects of radiation therapy that lead to symptoms of nausea, vomiting, and immunosuppression. In this study, we investigated the possibility that *L. ruthenicum* would be able to protect mice from injury due to radiation by reducing apoptosis and DNA damage. We hope *L. ruthenicum* can relieve the suffering of radiation therapy patients. According to these results, *L. ruthenicum* may have better therapeutic value in the clinic than previous products.

## 2. Materials and Methods

### 2.1. Ethical Statement

All procedures involved in the handling and care of animals were in accordance with the China Practice for the Care and Use of Laboratory Animals and were approved by the China Zoological Society (permit number: GB 14923-2010).

### 2.2. Reagents and Instruments

*L. ruthenicum* was purchased from Qinghai Jiukang Traditional Chinese Medicine Co., Ltd. (Xining, China). Acetonitrile and methanol, HPLC-grade, was obtained from Shandong Yuwang Industrial Co., Ltd. (Shandong, China), Chemical Branch. Amifostine (lot: 130306) was purchased from Tianjin Zhong Rui Pharmaceutical Share Co., Ltd. (Tianjin, China). Enzyme-linked immunosorbent assay (ELISA) kits to measure mouse caspase-3 (lot: 20141227.60325M), and mouse caspase-6 (lot: 20141227.60284M) were purchased from Beijing RigorBio Science Development Co., Ltd. (Beijing, China). Blood cell hemolysis reagent (lot: 2013111101), three classification probe cleaning fluid (lot: 2013112101), and dilution buffer for blood cell analyzer (M-23D, lot: 2013110701) were purchased from Shenzhen Mindray Bio Medical Electronic Share Co., Ltd. (Shenzhen, China). The remaining reagents were analytically pure, and the water used was purified. HPLC was performed using an Aglient 1200 High Performance Liquid Chromatograph (Agilent Co., Santa Clara, CA, USA); a medical electronic linear accelerator (23EX, Varian, Palo Alto, CA, USA); and a UV-2550 ultraviolet spectrophotometer (Shimadzu Co., Tokyo, Japan). A TGL-16 B high speed freezing centrifuge was obtained from the Shanghai Anting Scientific Instrument Factory (Shanghai, China). An RT-2100C Enzyme-labelled meter was sourced from Shenzhen Rayto Life Science Share Co., Ltd. (Shenzhen, China). Finally, the BC-2300quasi automatic three classification blood cell analyzer used was purchased from Shenzhen Mindray (Shenzhen, China). P53 mouse anti-rabbit monoclonal antibody, Universal PV9000 immunohistochemistry Kit, repair solution of citric acid, phosphate buffer and DAB chromogenic reagent as well as antibody dilution were purchased from Beijing Zhongshan Golden Bridge Biotechnology Co., Ltd. (Beijing, China).

### 2.3. Extraction of L. ruthenicum

The first 300 g of fruits of *L. ruthenicum* was extracted 20 times with 6000 mL water. The procedure was conducted in a 70 °C water bath in a dark room for 4 h using a 10 L beaker with its opening sealed by Parafilm. Second, vacuum concentration at 70 °C was conducted so that each milliliter of the decoction contained an extract of 400 mg of the crude drug. The extraction of *L. ruthenicum* was preserved at 4 °C in the dark, ready for experimental testing.

### 2.4. Proanthocyanidins B_2_ and Total Anthocyanin Detection

*L. ruthenicum* (10 g) was measured accurately into a 200 mL brown bottle placed in a 70 °C water bath in a dark room for 4 h. It was then cooled, and the mixture was filtered through a microporous membrane and then immediately subjected to high-performance liquid chromatography. The following chromatographic conditions were used to determinate Proanthocyanidins B_2_: column, Diamonsil 5 μL C18, (250 × 4.6 mm); detection wavelength, 280 nm; column temperature, 30 °C; sample load, 20 µL; flow rate, 1 mL/min and a gradient elution of mobile phase A (2% acetate) and mobile phase B (acetonitrile) (Table 1).

**Table 1:** Proanthocyanidins B_2_ samples separated using gradient elution chromatography.

Time (min)	0	15	40	45
2% Acetate (%)	92	88	75	60
Acetonitrile (%)	8	12	25	40

Total anthocyanins: *L. ruthenicum* (10 g) was measured accurately into a 200 mL brown bottle placed in a 70 °C water bath in a dark room for 4 h. It was then cooled, filtered, and samples (2 mL) were added to 48 mL KCL-HCL (0.2 mol/L KCL: 0.2 mol/L HCL = 25:67) in a volumetric flask (10 mL). The buffer solution was taken as zero, and the absorbance was measured at a wavelength of 526 nm. The procedure was repeated three times.

### 2.5. Animals and Experimental Treatments

A total of 180 4–6 week old male Kunming mice (25 ± 2 g) of SPF grade were provided by Gansu University of Traditional Chinese Medicine Animal Center (SCXK2011-0001, Gansu, China). The animals were adapted for a week at 23 ± 2 °C with a constant humidity of 55% ± 5% under a cycle of 12 h of dark, and given *ad libitum* access to water and food pellets. Ten animals were housed per cage with separated rooms to ensure each of them can be restrained in a single space so as to avoid restraint stress. All experiments were approved by the School of Medical Science, Medical College of Qinghai University and conducted according to good laboratory practice (GLP) for drugs.

Sixty mice were randomly divided into six groups: control group, model group, positive group (amifostine, 150 mg/kg, body weight/day), *L. ruthenicum* high dose (8 g/kg body weight/day, LH), *L. ruthenicum* middle dose (4 g/kg body weight/day, LM), and *L. ruthenicum* low dose (2 g/kg, body weight/day, LL) group used for the experiment on the third day after radiation. In the same way, another 120 mice were used for the experiments on the seventh and 14th days after radiation. Different doses of *L. ruthenicum* extract were administered intragastrically to the mice for 14 consecutive days. Model and control groups were orally administered with normal saline. The positive group was administered an intraperitoneal injection of amifostine. Before experiments, animals were immediately anesthetized via enterocoelia injection with sodium pentobarbital (50 mg/kg).

### 2.6. Irradiation

The Qinghai University Affiliated Hospital was used for the irradiation experiments. All mice, except the control group, were restrained in special boxes and exposed to 5.0 Gy total-body X-radiation at a dose rate of 300 cGy/min. The source-to-animal distance was 100 cm. Radiation time: 100 s.

### 2.7. Blood Cell Count

On the 3rd, 7th, and 14th day after radiation, respectively, 20 μL ocular blood was taken from all mice using a centrifuge tube with EDTA-2Na. Blood cell count (leucocytes-WBC, erythrocytes-RBC, hemoglobin-HGB and thrombocytes-PLT) was determined by use of a hemocounter.

### 2.8. Thymus and Spleen Index

On the 3rd, 7th, and 14th day after radiation, respectively, the thymus and spleen were removed from all mice. The thymus and spleen index was calculated by dividing organ weight by body weight (BW):

Thymus index (%) = thymus weight (g) / BW (g) × 100%

Spleen index (%) = spleen weight (g) / BW (g) × 100%

### 2.9. DNA Contents of Bone Marrow Cell

All mice, on the 3rd, 7th, and 14th day after radiation, respectively, were sacrificed by cervical dislocation. The bone marrow was taken from the whole femoral bone by flushing with 10 mL 0.005 mol/L CaCl_2_ until the femoral bone became white. The bone marrow was placed in a 5 mL centrifuge tube at −20 °C for 30 min, and then centrifuged at 2500 r/min for 15 min (12 cm rotor diameter). The supernatant was discarded and the sediment applied to 5 mL 0.2 mol/L HClO_4_ and fully mixed. Water bath heating lasted 15 min, followed by flowing water cooling. The bone marrow solution was filtered by filter paper and determined at 260 nm using an ultraviolet spectrophotometer.

### 2.10. Caspase-3 and Caspase-6 Contents

On the 3rd, 7th, and 14th day after radiation, respectively, blood was taken from all mice using a centrifuge tube. For this purpose, blood samples were taken from the eyeball of animals. The blood was centrifuged at 3000 r/min for 10 min to separate the serum. Caspase-3 and caspase-6 levels in the serum were detected using ELISA. In standard orifices, standard diluents and Str-HRP-Conjugate Reagent (50 µL of each) were added, which already contained combined biotin antibody. In turn, to sample orifices, 40 µL of sample, 10 µL of caspase-3 antibody, caspase-6 antibody, and 50 µL Str-HRP-Conjugate Reagent were added. The plate was covered, sealed, and incubated at 37 °C for 60 min with gentle shaking. The liquid was discarded, and the plate was spin-dried, washed using an automatic plate washer, and patted dry. Chromogen solution A and B (50 µL of each) was added to each well, mixed gently, and incubated for 15 min at 37 °C in the dark. The reaction was then stopped by the addition of 50 µL stop solution, and the optical density (OD) at 450 nm was measured within 15 min.

### 2.11. Immunohistochemistry

The small intestine tissues of mice were placed in 4% paraformaldehyde fixative at 4 °C overnight. The tissue was dehydrated and paraffin embedded. Microtome sections were obtained and mounted on slides. For immunohistochemistry, the slides were deparaffinized in xylenes and then rehydrated for 3 min each in 100% ethyl alcohol, 95% ethyl alcohol, 70% ethyl alcohol, 50% ethyl alcohol, and ddH_2_O. Antigen retrieval was accomplished by incubating slides in 10 mM sodium citrate and heating in a microwave oven on high for 2 min and on low for 7 min. The slides were cooled in sodium citrate solution for 20 min, washed in TBS-T (Tween) to permeabilize, and then incubated in 3% hydrogen peroxide in TBS for 15 min. The sections were blocked for 1 h in 10% serum (from host of secondary antibody) in 3% BSA-TBS at room temperature and incubated overnight in primary antibody (anti-P53) diluted 1:500 in the blocking solution. A ChemMate^TM^ Envision^TM^ Detection Kit was used for immunohistochemistry according to the manufacturer’s instructions. Immunohistochemical images were acquired on a DP71 microscope (Olympus, Tokyo, Japan) and DP CONTROLLER software.

### 2.12. Statistical Analysis

All quantitative data are expressed as mean ± SD deviations. The data were analyzed using one-way analysis of variance (ANOVA) in statistical package for social sciences (SPSS 17.0), and the differences between the means of two groups were compared using LSD tests. The results were considered to be statistically significant when *p <* 0.05.

## 3. Results

### 3.1. Proanthocyanidins B_2_ and Total Anthocyanidin Detection

**Figure 1:**
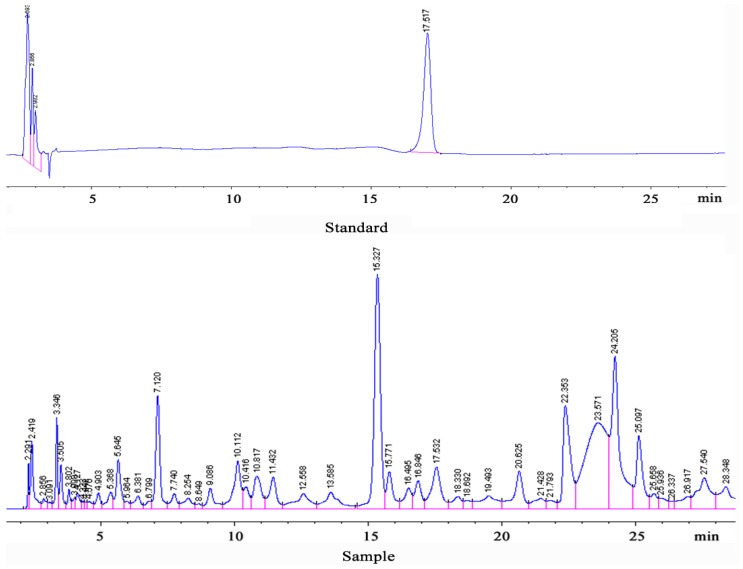
HPLC chromatograms of proanthocyanidins B_2_ in *L. ruthenicum*.

The proanthocyanidins B_2_ and total anthocyanidins in *L. ruthenicum* were 0.141 g/100 g (Figure 1), were 0.639 g/100 g, respectively.

### 3.2. Effect of Lycium ruthenicum Murr. on Body Weight of Irradiated Mice

**Figure 2:**
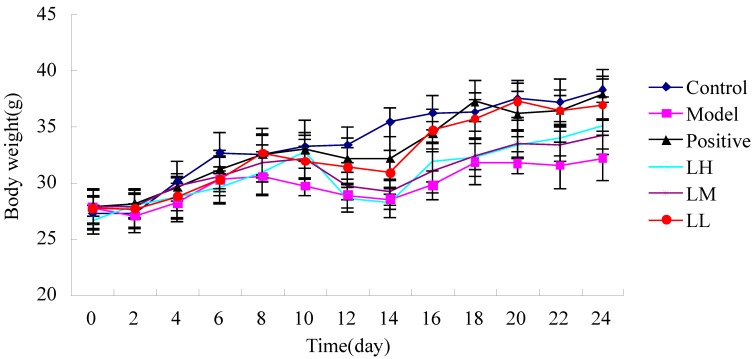
Effect of *Lycium ruthenicum* Murr. on body weight of irradiated mice.

Figure 2 shows that, except for control group, when mice were irradiated on the 10th day, others showed weight decreases compared with body weight before irradiation. The body weight of mice began to increase at six days after irradiation. However, the body weight increase of mice was accelerated by *L. ruthenicum* and *L. ruthenicum* could also prevent loss of body weight.

### 3.3. Effect of Lycium ruthenicum Murr. on the Hemogram of Mice after Radiation

**Figure 3:**
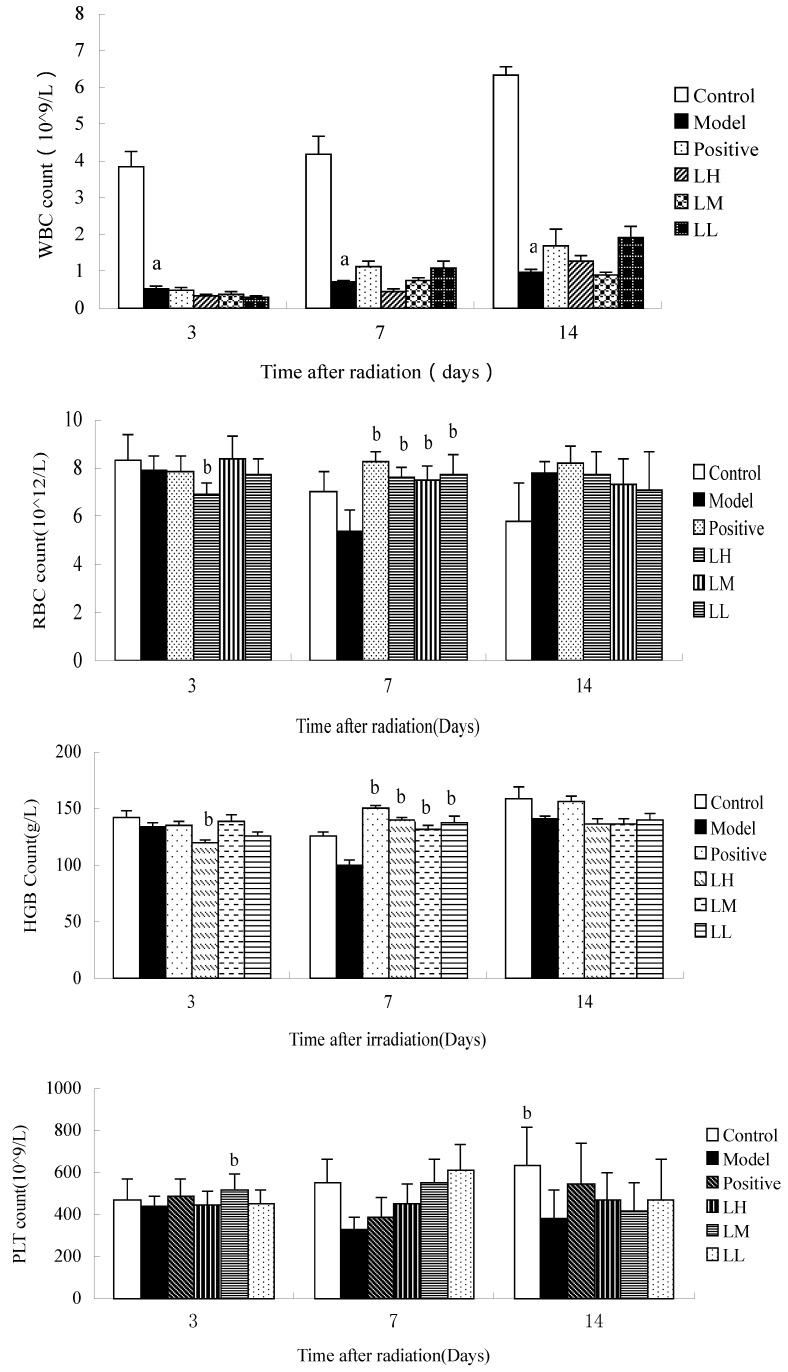
Effect of *Lycium ruthenicum* Murr. on the hemogram of mice after radiation. n = 10, mean ± SD. ^a^ *p <* 0.05 *vs.* control group, ^b^ *p <* 0.05 *vs.* model group.

Figure 3 shows that compared with the WBC count of the control group the WBC counts of mice in the model group on the 3rd, 7th, and 14th day after irradiation were reduced (*p <* 0.05). Compared with model, the LH group showed reduced RBC count at three days after radiation (*p <* 0.05)*.*

Seven days after irradiation, the RBC count of mice in positive group and LH, LM, LL group compared with model was increased (*p* < 0.05). The HGB count of mice in the LH group at three days after irradiation was reduced (*p* < 0.05), and HGB count in the other groups was higher than the model at seven days after irradiation. However, there was no significant difference in HGB count between the groups at 14 days after irradiation (*p* > 0.05). Fourteen days after irradiation, PLT count in the model was lower than control group (*p* < 0.05). Time after irradiation has a significant effect on the HGB and RBC count in each group (*p* < 0.05). The results indicated that *L. ruthenicum* treated irradiated groups did not show any significant dose-dependency.

### 3.4. Effect of Lycium ruthenicum Murr. on the Index of Thymus and Spleen of Mice after Radiation

**Figure 4:**
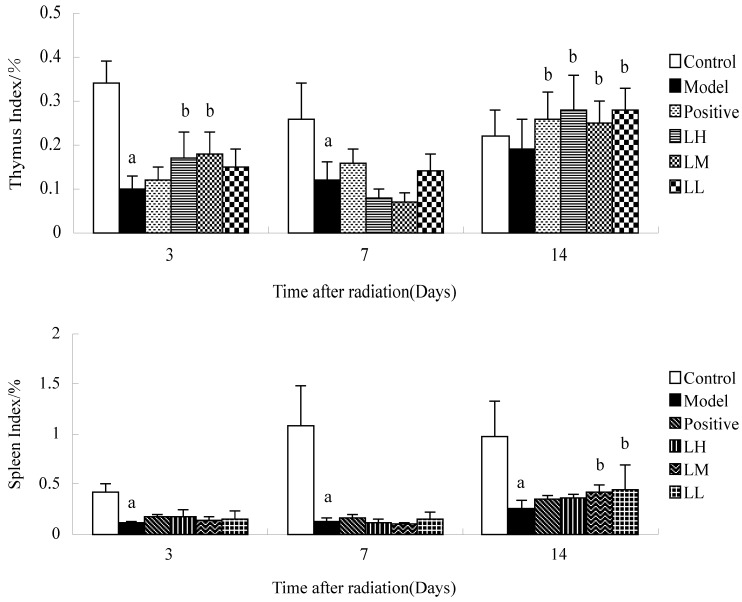
Effect of *Lycium ruthenicum* Murr. on the index of thymus and spleen of mice after Radiation. *n* =10, mean ± SD. ^a^ *p* < 0.05 *vs.* control group, ^b^ *p* < 0.05 *vs.* model group.

Figure 4 shows that the thymus and spleen index of mice in model group at three and seven days after irradiation compared with thymus and spleen index of control group was reduced (*p <* 0.05). Compared with the model, LH and LM groups showed increased thymus index at three and seven days after radiation (*p <* 0.05). Fourteen days after irradiation, the spleen index of mice in LM and LL group compared with model was increased (*p <* 0.05). Spleen index in the model was lower than control group at three, seven, and 14 days (*p <* 0.05). Time after irradiation has a significant effect on the thymus index and spleen index in each group (*p <* 0.05).

### 3.5. Effect of Lycium ruthenicum Murr. on the DNA Content of Mice after Radiation

**Figure 5:**
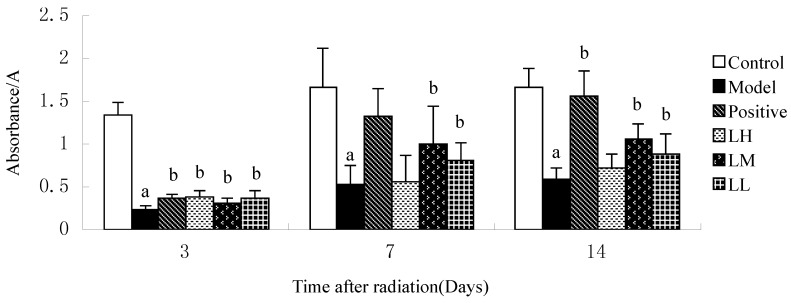
Effect of *Lycium ruthenicum* Murr. on the DNA content of mice after radiation. *n* = 10, mean ± SD. ^a^ *p* <0.05 *vs.* control group, ^b^ *p* < 0.05 *vs.* model group.

Figure 5 shows that the DNA contents of mice in model group at three days after irradiation compared with DNA contents of LH, LM, LL group was increased (*p <* 0.05). Compared with model, LM and LL group showed increased DNA contents at seven and 14 days after radiation (*p <* 0.05). The DNA contents of mice in model group at three, seven, and 14 days after irradiation compared with DNA contents of control group was increased (*p <* 0.05). Time after irradiation has a significant effect on the DNA contents in each group (*p <* 0.05).

### 3.6. Effect of Lycium ruthenicum Murr. on Caspase-3 and Caspase-6 of Mice after Radiation

**Figure 6:**
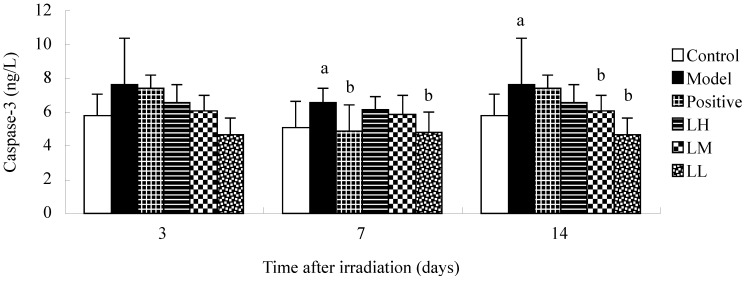

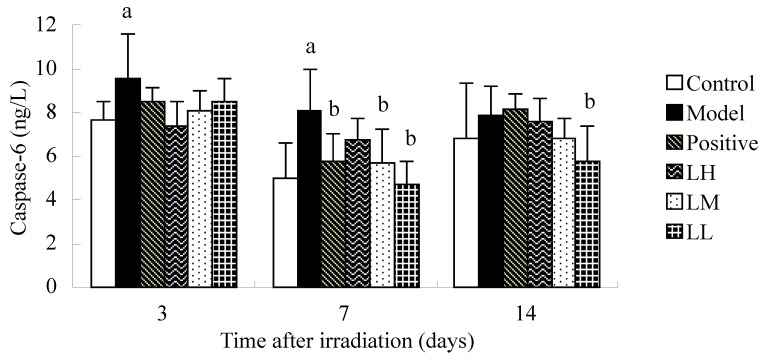
Effect of *Lycium ruthenicum* Murr. on the caspase-3 and caspase-6 of mice after radiation treatment. *n* = 10, mean ± SD. ^a^ *p* < 0.05 *vs.* control group, ^b^ *p* < 0.05 *vs.* model group.

The standard linear regression equations for caspase-3 and caspase-6 were Y = 0.0837X + 0.0287, *R*^2^ = 0.9990 and Y = 0.0678X + 0.0488, *R*^2^ = 0.9980, respectively. Figure 6 shows that there was no significant difference in the caspase-3 contents between the groups at three days after irradiation (*p >* 0.05). The caspase-3 contents of mice in positive group at seven days after irradiation compared with DNA contents of model group was reduced (*p <* 0.05). Caspase-3 in the model was lower than control group at seven and 14 days after irradiation (*p <* 0.05). Fourteen days after irradiation, the caspase-3 contents in LM and LL group was reduced compared with caspase-3 contents in the model (*p <* 0.05). Three days after irradiation, caspase-3 in model was lower than control group (*p <* 0.05). Compared with model, LM and LL and positive group showed reduced caspase-6 contents at seven days after radiation (*p <* 0.05). Caspase-6 in control group was higher than model group at seven days (*p <* 0.05). Fourteen days after irradiation, caspase-6 in LL group was lower than model group (*p <* 0.05). Time after irradiation has a significant effect on the caspase-3 and caspase-6 contents in each group (*p <* 0.05).

### 3.7. Effect of Lycium ruthenicum Murr. on the P53 of Mice after Radiation

**Figure 7:**
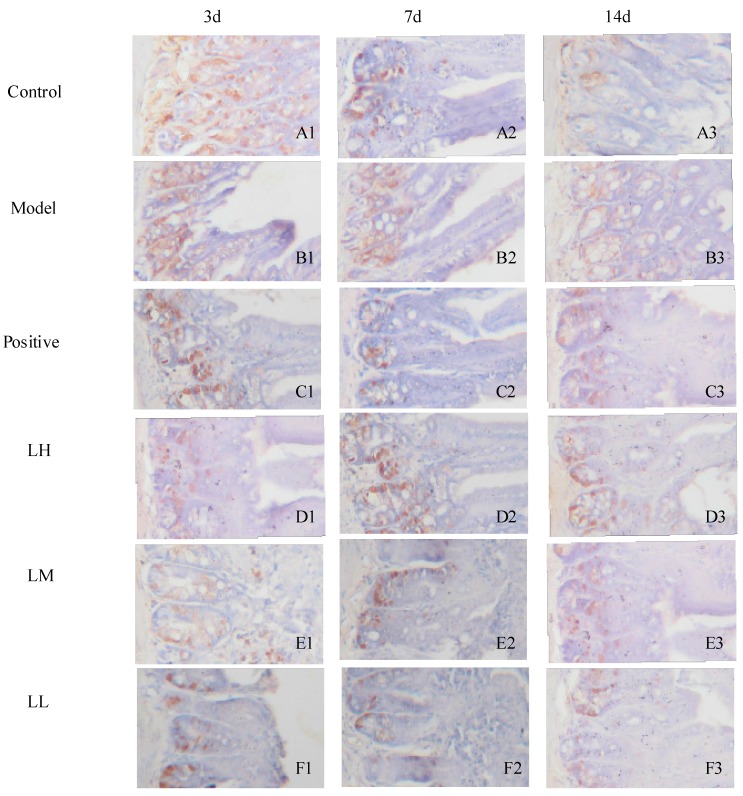
Expression of P53 in the intestinal epithelial cells (Obj. 20×).

Figure 7 shows *L. ruthenicum* had no significant effect on the P53 of mice after three days. However, LL group could significantly reduce P53 at seven days after radiation and LL and LM significantly reduced P53 at 14 days after radiation. The expression of P53 of model group gradually decreased over time.

## 4. Discussion

The blood system is the most sensitive target-organ of radiation. The reduction of leukocytes is the most classic indicator of radiation damage [17]. In this study, leukocytes were greatly reduced after irradiation. The erythrocyte, hemoglobin, and platelet counts of mice reached the lowest at seven days after irradiation. However, *L. ruthenicum* could tolerate the reduction of erythrocyte and hemoglobin at seven days after irradiation (*p <* 0.05).

As Figure 1 shows, leukocyte progressively increased as time went by. Furthermore, factorial analysis variance confirmed that the time after irradiation could significantly increase WBC count. Except for the leukocyte count in model group, erythrocyte, hemoglobin, and platelet numbers in all groups increased at first and then decreased to the lowest point at seven days after irradiation and significantly recovered at 14 days after irradiation.

Thymus and spleen are the most important hematopoietic organs. Our data showed that thymus and spleen index was reduced after irradiation (*p <* 0.05), which is consistent with other reports [18,19]. The thymus index at seven and 14 days after irradiation and the spleen index at 14 days after irradiation were increased by *L. ruthenicum* (*p <* 0.05)*.* All this showed that *L. ruthenicum* could have a certain protective effect in mice with thymus and spleen injury induced by radiation. Thymus and spleen index progressively increased as time went by. The comparison of spleen index between *L. ruthenicum* group and model showed no significant difference at three and seven days after irradiation (*p >* 0.05), however, spleen index significantly increased at 14 days after irradiation (*p* < 0.05)*.* This also confirmed that the recovery of radiation injury mice was accelerated by *L. ruthenicum.* Thymus and spleen are not only hematopoietic organs but also immunity peripheral organs, which represent immune function. The results indicated that the recovery of immune function of radiation injury mice was accelerated by *L. ruthenicum.* However, the mechanism of cell level is yet to be clearly defined.

Mature blood cell responses to ionization radiation exposure show that the function of the hematopoietic system could be damaged by radiation. The lack of blood cell resources, eventually, leads to a decrease in blood cell levels [20]. DNA of bone marrow is an important index that symbolizes the function of the hematopoietic system [21,22]. Our results showed that *L. ruthenicum* could significantly increase DNA contents in bone morrow and reduced caspase-3 and caspase-6 in the serum at seven and 14 days after irradiation (*p <* 0.05)*.* Caspase-3 and caspase-6 are apoptotic executioners of the caspase family that are both highly homologous. Caspase-3 could be activated by activated caspase-6. The activation of caspase-3 as an important member of the caspase family indicates that apoptosis had reached an irreversible stage [23,24]. The activated caspase-3 cuts PARP (poly ADP-ribose polymerase), which activated the important factors of DNA repair, thus achieving the goals of DNA repair [25,26,27]. The contents of caspase-3 is reduced by *L. ruthenicum*, which indicates that *L. ruthenicum* could resist the damage of DNA repair factors. Caspase-6 is unique in the caspase family in that it cuts lamina protein in the process of apoptosis, resulting in nuclear lamina damage so that condensed chromosomes finally lead to apoptosis [28,29,30]. *L. ruthenicum* could protect chromosomes by reducing the contents of caspase-6 and lamina protein. The DNA damage by radiation may result in the cell cancelation; in addition, the reduction of caspase-3 and caspase-6 would lead to the mass proliferation of tumor cells. The positive drug amifostine affects the cell apoptosis in double adjustment by increasing the activity of apoptosis factors to the cancer cells and inhibiting the activity of apoptosis factors to the normal cells [31,32]. It is observed that caspase-3 and caspase-6 in the positive drug group also declined, proving that *L. ruthenicum* could down-regulate the apoptosis of normal cells; hence, protect DNA and enhance the ability to resist radiation. The results of P53 is consistent with caspase. The P53 was increased after radiation and caused cellular apoptosis. However, the expression of P53 of *L. ruthenicum* group was decreased significantly; then *L. ruthenicum* could down-regulate the apoptosis of normal cells. In addition, in our pre-experiments, we found that *L. ruthenicum* can reduce the activity of antioxidant enzymes. *L. ruthenicum* contains an increased number of anthocyanins that could increase the activity of antioxidants. Free radicals are generated after radiation, that can attack the DNA and induce apoptosis, but in this study, as the figures show, *L. ruthenicum* can reduce the apoptosis after radiation. We infer that *L. ruthenicum* acts by directly quenching free radicals caused by radiation, but the concrete mechanism whereby it reduces the activity of antioxidant enzymes and whether it has a double adjustment effect needs further study.

## 5. Conclusions

We firstly found that *L. ruthenicum* has a certain protective effect against radiation injury in mice. In addition, we also reveal that *L. ruthenicum* can accelerate the recovery of the peripheral blood system and increase the DNA contents of bone morrow, as well as reduce the apoptosis of cells. It can also increase immunological function. Furthermore, we established HPLC conditions for the determination of proanthocyanidins B_2_ and detected its content in *L. ruthenicum*, providing a basis for the quality control of *L. ruthenicum*. In summary, the results of this study provided experimental data for the medical application of *L. ruthenicum*. Up till now, *L. ruthenicum* was not only a Chinese herb for traditional medicine, but has also been used as a unique nutritional food, which could be developed and utilized as an effective health product after radiation therapy.
